# Reconstructed and analyzed X-ray computed tomography data of investment-cast and additive-manufactured aluminum foam for visualizing ligament failure mechanisms and regions of contact during a compression test

**DOI:** 10.1016/j.dib.2017.11.072

**Published:** 2017-11-26

**Authors:** Kristoffer E. Matheson, Kory K. Cross, Matthew M. Nowell, Ashley D. Spear

**Affiliations:** a Department of Mechanical Engineering, University of Utah, Salt Lake City, UT, USA; b AMETEK Materials Analysis Division, Draper, UT, USA

## Abstract

Three stochastic open-cell aluminum foam samples were incrementally compressed and imaged using X-ray Computed Tomography (CT). One of the samples was created using conventional investment casting methods and the other two were replicas of the same foam that were made using laser powder bed fusion. The reconstructed CT data were then examined in Paraview to identify and highlight the types of failure of individual ligaments. The accompanying sets of Paraview state files and STL files highlight the different ligament failure modes incrementally during compression for each foam. Ligament failure was classified as either “Fracture” (red) or “Collapse” (blue). Also, regions of neighboring ligaments that came into contact that were not originally touching were colored yellow. For further interpretation and discussion of the data, please refer to Matheson et al. (2017) [1].

**Specifications Table**

**Table:** 

Subject area	Materials science
More specific subject area	*Cellular metals*
Type of data	*3D image reconstructions*
How data was acquired	*X-ray Computed Tomography using a Varian BIR 150/130 imaging system*
Data format	*Reconstructed and analyzed (compatible with Paraview 5.2.0)*
Experimental factors	*One aluminum foam specimen was manufactured using conventional investment casting techniques; the other two were created using laser powder bed fusion*
Experimental features	*Samples of each type of specimen were compressed and imaged at 2* *mm increments of displacement in an X-ray CT system*
Data source location	*Salt Lake City, Utah, USA*
Data accessibility	*National Institute of Standards and Technology (NIST) Materials Data Repository,*http://hdl.handle.net/11256/949

**Value of the Data**

• The data help to quickly identify patterns of failure among three different foam samples that were created using one of two manufacturing methods: investment casting or laser powder bed fusion
• The data make it simple to follow the complete evolution of damage for any of the foam samples, beginning with the uncrushed specimen all the way to the maximum compression level achieved during the test
• The data allow examination of the brittle or ductile behavior of the two different types of foam based on the ligament fracture or collapse patterns

## 1 Data

The data consist of three sets of Paraview state files and STL files, and each set corresponds to the compression test for a single foam specimen. In each set, there are seven displacement increments, and one Paraview state file / STL file combination is given for each displacement increment. The STL may be viewed on its own using any STL viewing software; however no regions will be highlighted. Paraview must be used in order for ligaments to be highlighted as described above. This can be done in Paraview by selecting **File** -> **Load State…** and then navigating to the location of the relevant.pvsm file. A Paraview dialog box will then open requesting the location of the corresponding .stl file. Navigate to the.stl file that accompanies the.pvsm file and click **OK**. The state files were created using Paraview 5.2.0 and might not be fully compatible with all versions of Paraview.

## 2 Experimental design, materials and methods

The experimental procedure used to generate the data reported here is described in detail in [1].

The sequence of STL files included in this data set for each of three aluminum foam samples was derived from in-situ imaging using a Varian BIR 150/130 X-ray CT imaging system. The 18.3 mm-long foam samples (one investment cast and two produced by laser powder bed fusion) were loaded in displacement control to a total displacement of 12 mm (approximately 66% of initial height). X-ray CT scan images were collected every 2 mm of displacement, during which the displacement was held constant. These images were reconstructed and segmented in Avizo.

The segmented data from Avizo was used to create STL files for each foam at each compression increment, which were then separately examined in Paraview. The different ligament failure modes were identified and highlighted. Ligament failure was classified as having experienced either “Fracture”, where ligaments appeared to exhibit some amount of brittle fracture after the previous compression increment, or as having experienced “Collapse”, which includes buckling and/or bending of ligaments. The “Fracture” ligaments were highlighted in red and the “Collapse” ligaments were highlighted in blue. Ligaments that remained the same or mostly the same between the current increment and the previous one were left unhighlighted, even if they had previously experienced some type failure in a previous increment. Also, regions of neighboring ligaments that came into contact that were not originally touching in the pristine foam were colored yellow.

The reader is strongly encouraged to see reference [1] for a more in-depth description of the experimental procedure and discussion of results.
